# Functional Brain Network Predictors of Abstinence Treatment Outcomes in Methamphetamine Use Disorder

**DOI:** 10.1002/cns.70990

**Published:** 2026-06-17

**Authors:** Yanyao Du, Shiqi Di, Na Luo, Wenhan Yang, Weiyang Shi, Zhengyi Yang, Ming Song, Huiting Zhang, Jun Zhang, Tianzi Jiang, Jun Liu

**Affiliations:** ^1^ Department of Radiology The Second Xiangya Hospital of Central South University Changsha Hunan China; ^2^ Brainnetome Center Institute of Automation, Chinese Academy of Sciences Beijing China; ^3^ School of Artificial Intelligence University of Chinese Academy of Sciences Beijing China; ^4^ MR Research Collaboration Siemens Healthineers Ltd. Wuhan China; ^5^ Hunan Judicial Police Academy Changsha Hunan China; ^6^ Center for Excellence in Brain Science and Intelligence Technology Institute of Automation, Chinese Academy of Sciences Beijing China; ^7^ Xiaoxiang Institute for Brain Health and Yongzhou Central Hospital Yongzhou Hunan China; ^8^ Clinical Research Center for Medical Imaging in Hunan Province The Second Xiangya Hospital of Central South University Changsha Hunan China; ^9^ Department of Radiology Quality Control Center The Second Xiangya Hospital of Central South University Changsha Hunan China

**Keywords:** brain imaging, craving, functional connectivity networks, long‐term methamphetamine abstinence, machine learning, predictive markers

## Abstract

**Background:**

Methamphetamine (MA) use poses a serious threat to community safety and public health. Despite the existence of some treatment modalities, relapse rates remain high and the effectiveness of these treatments varies among individuals. Identifying behavioral, neuroimaging, and gene expression biomarkers associated with treatment efficacy can enhance our multiscale understanding of the neurobiological mechanisms underlying individualized responses to abstinence‐based treatments. This approach has the potential to advance the development of personalized or innovative therapeutic strategies.

**Methods:**

Our study included 82 MA users and 68 healthy controls (HCs). Demographic information, craving scale scores, MA use assessment, and MRI scans were collected from the MA group prior to treatment. All MA users underwent abstinence‐based treatment, during which they refrained from using MA and received only basic medical care and education on abstinent rehabilitation. Following long‐term abstinence‐based treatment, craving scale scores were reassessed. A reduction in craving scale scores greater than 30% was defined as the responders. Similarly, demographic information and MRI scans were collected from the 68 HCs. We calculated whole‐brain functional connectivity based on fMRI data and applied principal component regression (PCR) with leave‐one‐out cross‐validation to identify response network patterns predictive of abstinence response scores. Furthermore, we evaluated the stability of the predictive models from multiple perspectives. Network strength of the identified response network was then compared to that of HCs to assess its clinical relevance. We also assessed the efficacy of network strength in making binary predictions. Finally, we combined the discovered brain patterns with the Allen Human Brain Atlas data to explore the genetic basis associated with the identified response network.

**Results:**

Among the 82 MA users, 39 were responders and 43 were non‐responders. The 68 HCs had a mean age of 40.1 years, with 46 males. PCR identified a stable MA response network pattern, characterized by network connections positively associated with attention regulation and executive control abilities (within visual, between frontoparietal and default mode, and between visual and dorsal attention), as well as negative network connections associated with emotion regulation and behavioral automatization (within somatomotor, between somatomotor and default mode, and between default mode and ventral attention). HC exhibited moderate levels of network strength between responders and non‐responders. The identified network pattern demonstrated efficacy in individual‐level binary predictions. This neuroimaging pattern was further associated with synaptic signaling and inhibitory neurons.

**Conclusions:**

Together, our results not only provide new neuroimaging markers for predicting personalized treatment response, but also reveal the underlying neurobiological mechanisms associated with abstinence response, providing potential regulatory targets for addiction treatment.

## Graphical abstract

Baseline whole‐brain functional connectivity predicted 1‐year abstinence response in methamphetamine use disorder at the individual level. Multi‐level network analyses revealed distinct response‐related connectivity patterns involving control‐related circuits. Transcriptomic association analyses further linked these predictive networks to neurobiological pathways related to synaptic signaling and neurotransmitter function.

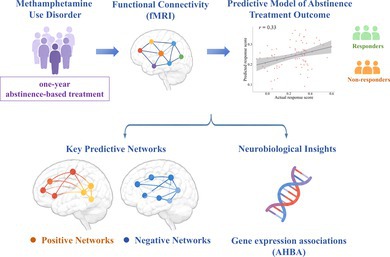

## 1 Introduction

Methamphetamine (MA) is a highly addictive and relapsing nervous system stimulant [1, 2, 3], and its use is steadily increasing each year [4]. Neuropsychiatric symptoms and infectious diseases caused by MA use pose great challenges to community safety and public health [2]. Despite the availability of both noninvasive and invasive interventions and treatments, such as psychotherapy [5], pharmacological interventions [6], and deep brain stimulation therapy [7], these methods exhibit limited efficacy in reducing relapse rates and show significant interindividual variations in effectiveness [5, 6, 8, 9]. More targeted treatment strategies are needed to reduce relapse rates, but current studies lack reliable imaging predictors of the MA abstinence response. Therefore, constructing predictive models capable of identifying individualized treatment outcomes based on neuroimaging is of paramount importance. This endeavor holds promise for elucidating the potential neurobiological mechanisms underlying differences in abstinent outcomes and for guiding future personalized interventions.

Current research on addiction prediction and the exploration of potential neuroimaging biomarkers predominantly employs regression or correlation analyses to identify brain regions associated with abstinence [10, 11, 12]. Some studies have also relied on region‐of‐interest (ROI) analyses based on prior hypotheses [13, 14]. However, the relationship between addictive neural activity and behavior is often intricate, involving complex interconnections and network‐level activities across multiple brain regions. Traditional approaches may fail to effectively capture these intricate patterns. Furthermore, several studies have employed whole‐brain functional connectivity analyses to identify potential response predictors for traditional substances, such as opioids and cocaine [15, 16]. However, research on abstinence‐related predictive factors specific to MA remains scarce. In this study, targeting the neuroimaging‐based prediction of MA abstinence responses, we utilized principal component regression (PCR), a method widely applied in neuroscience prediction, which serves not only as a predictive tool but also as a means to identify potential intervention targets [17]. Our research adopts a fully data‐driven approach based on whole‐brain functional connectivity to construct PCR models [18, 19]. In this way, we can identify whole‐brain connectivity patterns predictive of MA abstinence responses and map these patterns to specific brain regions and networks.

Previous studies have primarily interpreted brain regions or regional indicators derived from predictive models based on their known functional characteristics, aiming to explore the potential mechanisms underlying abstinence behaviors [12, 15, 16, 20]. While this single‐dimensional functional analysis approach has provided some insights into the functional roles of brain regions associated with abstinence, it often overlooks deeper biological underpinnings, limiting our understanding of the neurobiological mechanisms of addiction and abstinence. The Allen Human Brain Atlas (AHBA) offers gene expression data across different brain regions [21, 22, 23]. By correlating the identified functional connectivity patterns with the gene expression data, we aim to uncover the possible gene regulatory mechanisms underlying the brain patterns associated with addiction prediction.

In this study, we applied PCR to resting‐state functional MRI data obtained before long‐term abstinence‐based treatment in MA users. We developed a predictive model for long‐term abstinence outcomes and identified response networks associated with abstinence prediction. Multilevel analyses were conducted to investigate neuroimaging patterns, including brain connections, brain networks, and network strengths in MA‐addicted individuals compared to healthy controls (HCs). Furthermore, we evaluated the ability of identified networks to discriminate between responders and non‐responders in binary prediction. Finally, we integrated the AHBA dataset to perform multiscale analyses, aiming to uncover the potential underlying neurobiological mechanisms.

## 2 Methods

### 2.1 Participants

**FIGURE 1:**
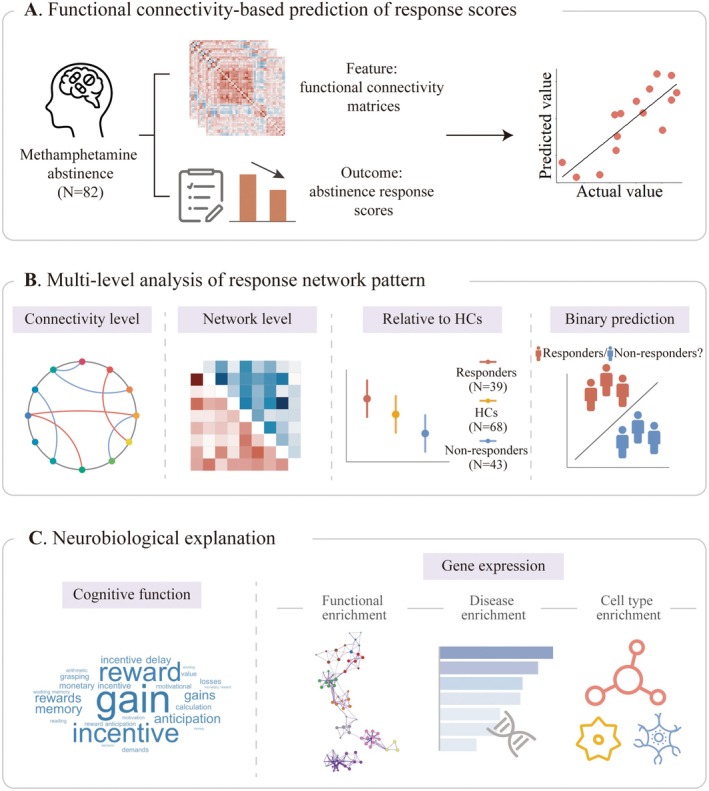
Overview of the analysis pipeline. (A) Functional connectivity‐based prediction of response scores. Pre‐abstinence neuroimaging data and relevant phenotypic information before and after abstinence were obtained from 82 methamphetamine‐dependent individuals. Predictive models were developed to predict abstinence response scores based on baseline whole‐brain functional connectivity. (B) Multi‐level analysis of response network pattern. Utilizing the weight map obtained from the predictive model, we examined the network pattern influencing the degree of abstinence response at both connectivity and network levels. The weight map was projected to healthy control (HC) subjects to assess response network strength relative to HCs. Furthermore, the same network pattern was applied to predict responders and non‐responders in a binarized manner. (C) Neurobiological explanation. We conducted further analyses to explore cognitive function and gene expression associated with the identified response network pattern. Gene lists linked to the identified pattern were annotated through functional, disease‐related, and cell type enrichment analyses.

The experimental design is shown in Figure 1. MA withdrawal subjects were recruited from drug rehabilitation centers in Changsha, Zhuzhou, and Yueyang (Hunan Province, China). The inclusion criteria for MA were as follows: (a) positive urine test results for only MA and negative test results for other substances; (b) meeting the addiction diagnostic criteria of the Diagnostic and Statistical Manual of Mental Disorders, 5th Edition (DSM‐V); and (c) having normal vision and hearing and being right‐handed. In addition, a group of HCs with negative urine results for all substances was included for comparative purposes. The exclusion criteria for the two groups were as follows: (a) any MRI contraindications; (b) brain structural abnormalities; and (c) a history of metabolic disorders or personal/family history of psychiatric conditions (e.g., anxiety, depression, or sleep disorders); and (d) failed to complete baseline MR examination or two questionnaire assessments. A total of 82 MA users and 68 HC participants were included.

All subjects in both the MA and HC groups underwent baseline MR examinations and completed several questionnaires, including demographic data, the Fagerstrom Test for Nicotine Dependence (FTND) [24], and the Alcohol Use Disorder Identification Test (AUDIT) [25]. Prior to abstinence, MA users were assessed for MA use patterns and completed the initial Methamphetamine Craving Questionnaire (MCQ). Subsequently, all MA participants underwent the same 1‐year abstinence‐based treatment program in drug rehabilitation centers, during which they received standardized medical care, MA‐related rehabilitation education, and structured physical activities to support their recovery. At the 1‐year follow‐up, the MA group underwent a second MCQ assessment to evaluate craving changes. The MCQ were modified from existing drug craving questionnaires and validated specifically for MA use disorders [26, 27, 28, 29]. The questionnaire includes four factors: intention to use, drug craving, anticipation of positive outcomes, and lack of self‐control, with a total of 25 items. The rate of change in craving scores before and after 1 year of abstinence [(Craving_first − Craving_second)/Craving_first] was computed as the response score. After completion of the one‐year abstinence‐based treatment program, participants were classified according to the degree of craving‐score reduction. Subjects with a response score higher than 30% were operationally classified as responders, whereas those with a response score of 30% or lower were classified as non‐responders. This threshold was used only for secondary analyses requiring dichotomization into responders and non‐responders, including group‐wise response network strength comparisons and binary prediction, while the primary predictive analysis was conducted using the continuous response score.

### 2.2 MRI Data Acquisition and Processing

All images were acquired using a 3 T MRI scanner (MAGETOM Skyra, Siemens Healthcare, Erlangen, Germany) equipped with a 32‐channel head coil. The MRI protocol comprised T1‐weighted imaging, T2‐weighted imaging, three‐dimensional magnetically prepared rapid acquisition gradient echo (3D MPRAGE) sequences, and rs‐fMRI. The parameters for 3D MPRAGE scanning were set as follows: 176 sagittal slices, repetition time = 1450 ms, echo time = 2.03 ms, flip angle = 30°, voxel size = 1 × 1 × 1 mm [3], slice thickness = 1 mm, and field of view = 256 × 256 mm [2]. For rs‐fMRI, the parameters were as follows: 36 axial slices, thickness = 4 mm, field of view = 220 × 220 mm [2], repetition time = 2000 ms, echo time = 30 ms, flip angle = 80°, and 225 volumes.

The rs‐fMRI data underwent preprocessing using statistical parametric mapping and GRETNA (V2.0) [30] based on MATLAB 2016b with a series of steps: conversion from DICOM to NIfTI format, exclusion of the initial 10 time points, identification and exclusion of participants with excessive head movement, normalization to the Montreal Neurological Institute space utilizing the DARTEL registration method, regression to remove nuisance covariates, band‐pass filtering (0.01–0.08 Hz), and calculation of functional connectivity based on the Brainnetome atlas with 246 brain regions.

### 2.3 Construction of Predictive Models

In this analysis, we employed PCR to construct predictive models for individual responses to abstinence. The input features for the predictive models comprised whole‐brain functional connectivity matrices alongside abstinence response scores. To avoid circularity bias and identify optimal features, a nested cross‐validation approach was adopted [31, 32]. Specifically, in the outer loop, leave‐one‐out cross‐validation (LOOCV) was employed. Each iteration involved using all participants except one as the training set to predict the response value of the “left‐out” participant, and this process was repeated for each participant. Model performance was assessed using Pearson's correlation between the predicted and actual values. Within each outer loop training set, an inner loop cross‐validation procedure was performed to optimize the number of retained principal components (PCs) for PCR. Specifically, the optimal number of PCs was selected based on the configuration yielding the best prediction performance (Pearson's correlation between the predicted and actual values) in the inner loop. The selected number of PCs was then used to construct the model in the corresponding outer loop iteration. Across all iterations, the retained PCs explained 87.35% ± 2.61% of the variance in the original imaging features.

The significance of the prediction accuracy was evaluated using a permutation test. Through random shuffling of the correspondence between whole‐brain functional connectivity matrices and response values, the null distribution for the significance test was generated by repeating the prediction analysis on the shuffled data 5000 times. The *p*_perm_ value was calculated as (sum(*r*_perm_ > *r*_true_) + 1)/5001, where *r*_perm_ represents correlation coefficients obtained from permuted data and *r*_true_ represents the correlation coefficient from the original data.

### 2.4 Connectivity Contributions and Network Anatomy

The extent to which each connectivity contributed to the predictive model was quantified using model weights. The average weights from 82 iterations were utilized to provide an aggregate characterization of connectivity‐level contributions within the predictive model. At the network level, the 246 atlas‐defined brain regions were grouped into eight networks, encompassing seven networks delineated by Yeo et al. [33] and an additional subcortical network (detailed information can be found in Table S1). Subsequently, the weight of a connection between a pair of networks was determined as the sum of the weights of all connections between them, normalized by the total number of connections within the network pair [17, 34]. At the node level, the representation of weight for each brain region was derived by summing the weights of all connections linked to this region. This node‐level weight map was further leveraged to decode relevant cognitive functions. Specifically, the Neurosynth database (https://neurosynth.org/) [35] was applied to decode cognitive terms by assessing the similarities between the identified weight map and brain activation maps. This database compiles results from more than 15,000 published functional MRI studies by high‐frequency keyword searches. By leveraging the Neurosynth database and focusing on cognitive and behavioral terms, we conducted a functional meta‐analysis to decode the relevant terms and retained the most strongly associated terms.

### 2.5 Model Stability Evaluation

A series of analyses were conducted to assess the stability of the predictive models from various perspectives. First, we calculated inter‐correlations among the weights obtained from 82 models to evaluate the stability of the predictive weights. Second, the correlation between bootstrapped mean weights (with 5000 iterations) and the mean weights derived from the PCR models was also calculated to assess weight stability. The bootstrap test was conducted through random sampling of participants with replacement, with a predictive model constructed based on bootstrap samples for each iteration. Additionally, to assess the stability of the identified predictive patterns across different modeling approaches, partial least squares regression (PLSR), another widely used predictive model [36, 37], was employed to perform identical predictions. The correlation between the mean weights of the PLSR models and the mean weights of the PCR models was examined.

### 2.6 Binary Prediction

To validate the efficacy of the predictive model for clinical application, we further focused on its capacity to provide binary outcome predictions at the individual level. Utilizing response scores with a threshold of 30%, we partitioned MA‐dependent individuals into two groups: responders and non‐responders, where group labels served as the basis for binarized predictions. Subsequently, a 10‐fold cross‐validation technique was employed for classification analysis. To ensure the robustness of the results, we iteratively repeated the prediction process 1000 times using shuffle‐split techniques.

### 2.7 Transcriptomic Analysis

The Allen Human Brain Atlas (AHBA) dataset (https://human.brain‐map.org) [23] was utilized in this analysis. It included brain‐wide gene expressions obtained from six postmortem brains (age: 24–57 years; male/female: 5/1), comprising 3702 spatially distinct samples distributed across nearly the entire brain. Employing the abagen toolbox [38], we performed standard preprocessing procedures, including probe‐to‐gene reannotation, intensity‐based probes filtering, selection of representative probes, sample‐to‐region assignment, and normalization of expression values. Since 11 of the 246 brain regions defined by the Brainnetome Atlas did not contain any tissue samples, the final gene expression matrix included 235 regions and 15,605 genes. To explore the association between gene expression and the predictive FC pattern, we initially computed co‐expression matrices for each gene. Specifically, for a given gene (denoted as gene a), the (i,j) element of the corresponding co‐expression matrix Ca was calculated as the product of the expression values of gene a in brain regions i and j. Subsequently, the Pearson correlation coefficient was computed between the gene co‐expression pattern and the functional connectivity (FC) pattern for each gene (represented by average weights). A higher absolute value of the correlation coefficient indicated greater relevance of the gene to the identified FC pattern. Two gene lists were generated based on highly positive (top 5%) and highly negative (bottom 5%) correlations for further enrichment analysis. Gene lists derived with a threshold of 10% were also analyzed for enrichment.

Utilizing Metascape analysis [39], we conducted Gene Ontology (GO) biological process, Kyoto Encyclopedia of Genes and Genomes (KEGG) pathway, and DisGeNET enrichment analyses for the identified gene lists. All genes within the genome were used as the background for enrichment analysis. Furthermore, according to Seidlitz et al. [40], cell types were reorganized into seven canonical classes: astrocytes, endothelial cells, microglia, excitatory neurons, inhibitory neurons, oligodendrocytes, and oligodendrocyte precursor cells. Our identified gene lists overlapped with the gene list specific to each of the seven cell types separately to evaluate enrichment within each cell type. The ratio of overlapping genes to the total number of genes in our gene lists for each cell type was calculated. Permutation tests were then conducted with false discovery rate (FDR) correction to determine the significance level [40].

## 3 Results

### 3.1 Demographic Characteristics

Our study included 82 MA users (mean age 34.43 ± 8.82 years; 58 males, 71%), among whom 39 were abstinence responders (mean age 35.75 ± 7.86 years; 37 males, 95%; first craving scale score 67.03 ± 14.48; second craving scale score 41.82 ± 10.15), and 43 were non‐responders (mean age 33.26 ± 9.54 years; 21 males, 49%; first craving scale score 56.02 ± 14.32; second craving scale score 47.84 ± 8.31). Except for gender, there were no significant statistical differences between the responder and non‐responder groups in age, education level, nicotine, alcohol, and MA use history, or abstinence duration (Table 1). The study also included 68 healthy controls (mean age 40.10 ± 9.51 years; 46 males, 68%), with detailed statistical information on the MA and HC groups provided in Table S2. The comparisons between MA responders and the HC group, as well as between MA non‐responders and the HC group, were presented in Table S3.

**TABLE 1:** Demographic characteristics of included MA.

Characteristic	MA (*N* = 82)	Responders (*N* = 39)	Non‐responders (*N* = 43)	t/χ^2^/Z	*p*
Age (years)^a^	34.43 ± 8.82	35.75 ± 7.86	33.26 ± 9.54	1.268	0.209
Gender (male/female)^b^	58/24	37/2	21/22	18.771	< 0.001*
Education (years)^a^	9.43 ± 2.61	9.31 ± 2.33	9.53 ± 2.87	−0.391	0.696
Nicotine use (yes/no)^b^	75/7	34/5	41/2	1.748	0.186
FTND^a^	4.56 ± 2.09	4.49 ± 2.16	4.63 ± 2.05	−0.302	0.764
Alcohol use (yes/no)^b^	46/36	19/20	27/16	1.649	0.266
AUDI^c^	4.52 ± 5.92	0.00 (0.00, 6.00)	3.00 (0.00, 8.00)	−1.078	0.281
Handedness	82 R	39 R	43 R	—	—
Age of first use (years)^a^	26.95 ± 7.33	27.85 ± 6.64	26.14 ± 7.90	1.053	0.295
Duration of drug use (years)^a^	5.04 ± 4.27	5.59 ± 4.18	4.53 ± 4.34	1.118	0.267
Dosage of drug use (g/day)^a^	0.45 ± 0.33	0.41 ± 0.26	0.49 ± 0.38	−1.211	0.229
Abstinence period (days)^a^	342.66 ± 103.49	361.74 ± 82.72	329.11 ± 115.16	1.330	0.188
Frequency of abstinence attempts^b^				0.063	0.802
First abstinence attempt	62	29	33	—	—
Two or more abstinence attempts	20	10	10	—	—
First craving scores^a^	61.26 ± 15.34	67.03 ± 14.48	56.02 ± 14.32	3.456	0.001*
Second craving scores^a^	44.98 ± 9.66	41.82 ± 10.15	47.84 ± 8.31	−2.920	0.005*

*Note:* A significant level was set at *p* < 0.05. Data superscripted with asterisks (*) indicate significant differences between responders and non‐responders.

^a^ Two‐sample *t*‐test.

^b^ Chi‐squared test.

^c^ Two‐sample Wilcoxon‐Mann–Whitney U test. MA, methamphetamine user. N, number of subjects. FTND, Fagerstrom Test for Nicotine Dependence. AUDIT, Alcohol Use Disorders Identification Test.

### 3.2 Associations Between Clinical Variables and Abstinence Response

Utilizing Pearson's correlation analysis, we investigated potential associations between baseline clinical variables (duration of use, age at first use, dosage, FTND and AUDIT scores) and abstinence response scores. No statistically significant associations were detected between these baseline clinical variables and abstinence response scores (duration of use: *r* = 0.13, *p* = 0.25; age at first use: *r* = 0.17, *p* = 0.13; dosage of use: *r* = −0.11, *p* = 0.31; FTND: *r* = −0.04, *p* = 0.70; AUDIT: *r* = −0.12, *p* = 0.28).

### 3.3 Predicting Response Score

**FIGURE 2:**
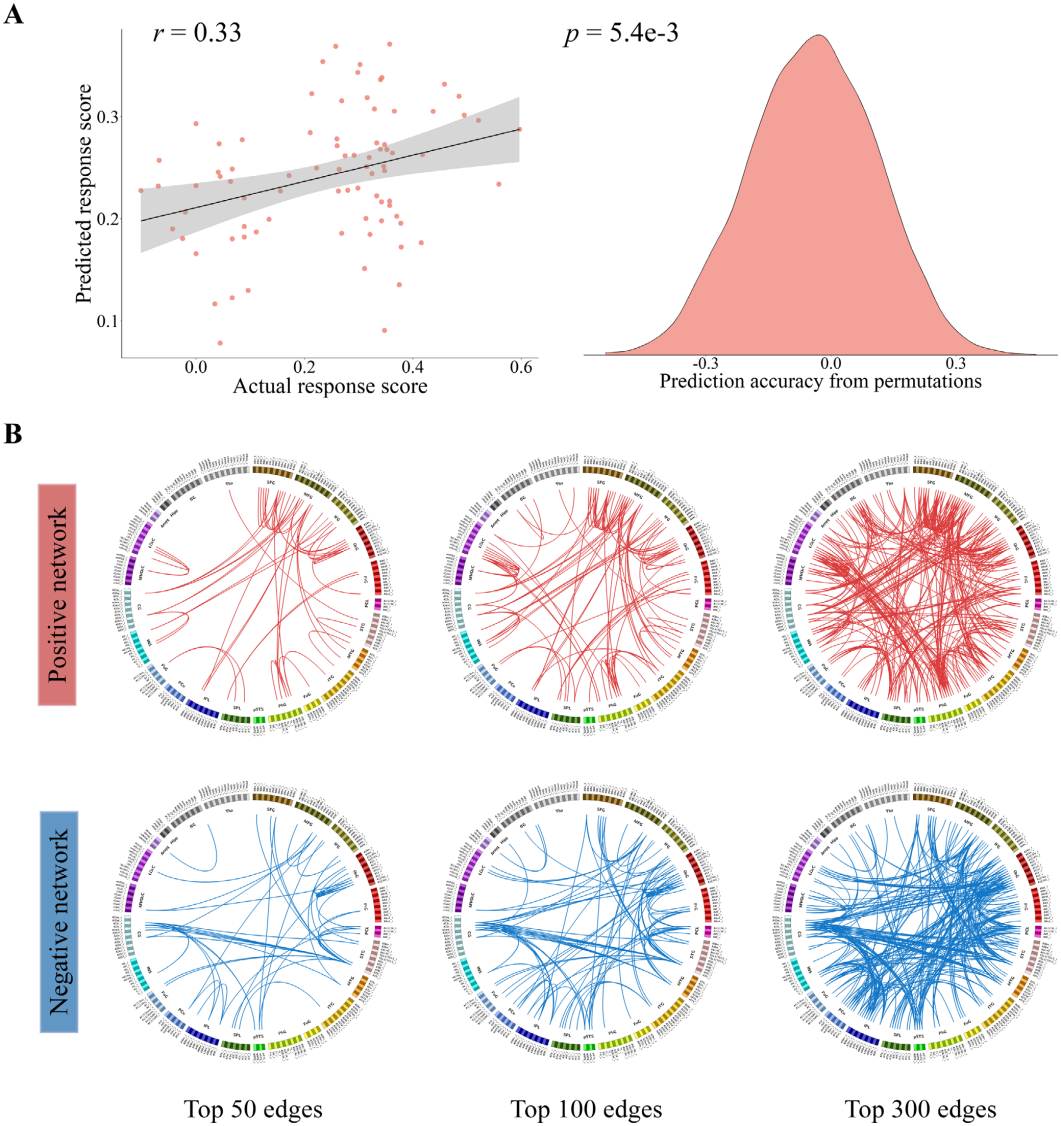
Predictive performance and response network. (A) Predictive performance using the PCR model. The left panel illustrates the correlation between actual and predicted response scores. The right panel depicts the distribution of prediction accuracies derived from 5000 permutation tests. (B) Connections in the identified response network that exhibit top contributions to the prediction. The positive network comprises connections with positive weights, while the negative network encompasses connections with negative weights.

Utilizing pre‐treatment whole‐brain functional connectivity data from MA users (*N* = 82), a PCR model was generated that could successfully predict the abstinence response scores (*r* = 0.33, permutation *p* = 5.4e‐3) (Figure 2A). In addition, Figure S1 presents the residual plot, in which the residuals were randomly distributed around zero, indicating the absence of obvious systematic bias. The mean residual was close to zero (mean residual = 0.0014), no significant heteroscedasticity was detected using the Breusch‐Pagan test (*p* = 0.22), and no extreme outliers were identified, further supporting the robustness of the predictive model.

The predictive response network was identified based on the model weights derived from PCR. Figure 2B summarizes the connections in the response network that contributed most significantly to the predictive model. In the positive network characterized by positive weights, connections involving regions such as the superior frontal gyrus (SFG), middle frontal gyrus (MFG), orbital gyrus (OrG), parahippocampal gyrus (PhG), and lateral occipital cortex (LoCC) exhibited prominent contributions. In contrast, connections involving the orbital gyrus (OrG), superior temporal gyrus (STG), paracentral lobule (PCL), posterior superior temporal sulcus (pSTS), and cingulate gyrus (CG) contributed more in the negative network. Furthermore, lesion analysis conducted by eliminating edges with the greatest contributions revealed a decrease in predictive performance with an increasing number of eliminated edges. Eliminating the top 300 edges from both the positive and negative networks resulted in an inability to predict abstinence response, underscoring the pivotal role of the identified response network in the prediction task (Figure S2A,B).

### 3.4 Network Anatomy

**FIGURE 3:**
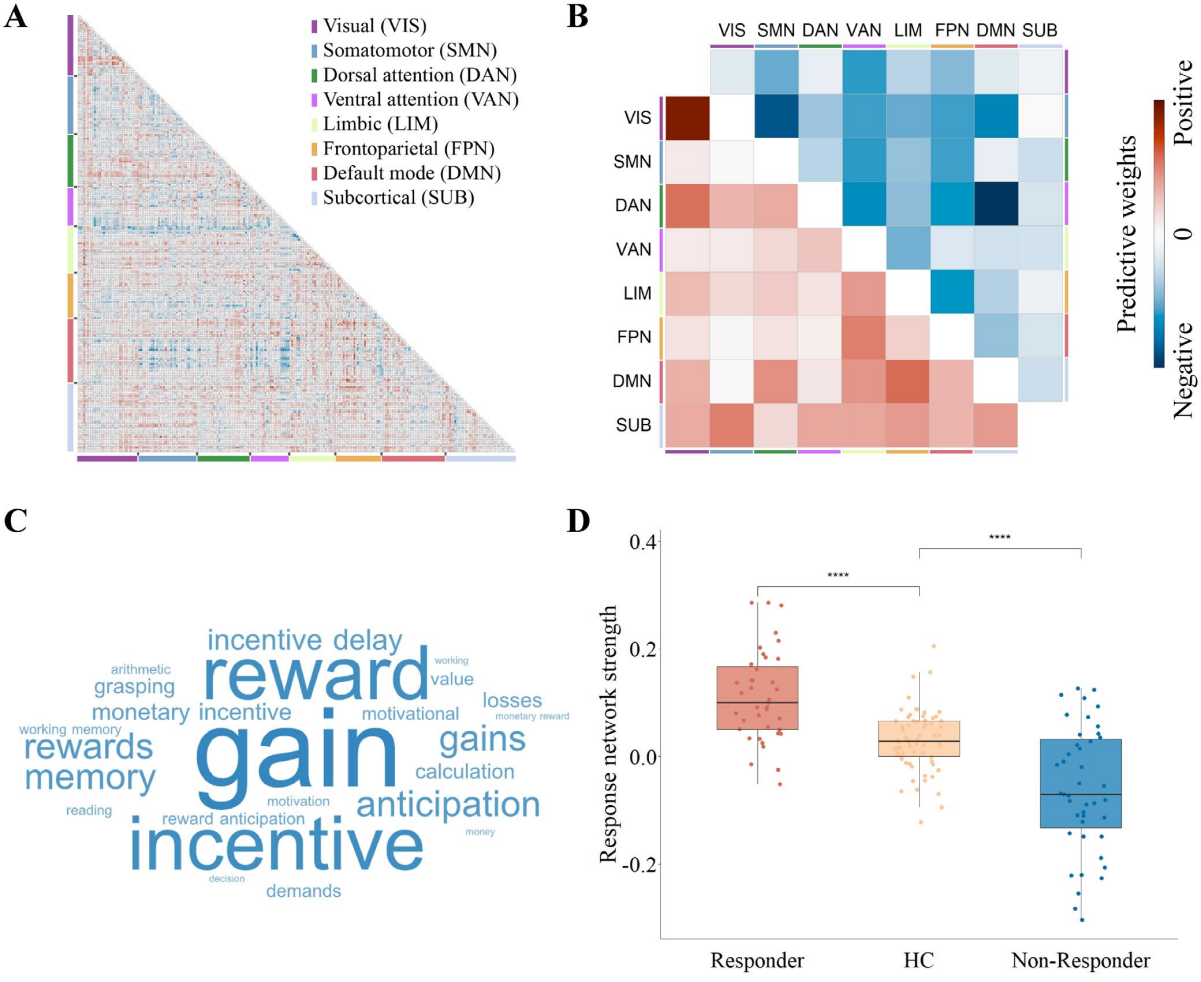
Response network pattern analysis. (A) The raw prediction weights of the model averaged across 82 iterations. Brain regions were reordered based on their respective network assignments. (B) Predictive weight characterization at the network level. The weight between a pair of networks was defined as the average of the corresponding weights of all connections in the pair. Separate calculations were performed for positive and negative weights. (C) Cognitive terms associated with brain regions exhibiting higher predictive power. Font sizes are scaled to reflect the correlation values of the respective cognitive terms. (D) Response network strength relative to HCs. Response network strength was determined by mapping the model weights to individual whole‐brain functional connectivity. The significance of differences was indicated by asterisks (*p* < 0.0001).

To facilitate the interpretation of the identified response network pattern (Figure 3A), we computed a network‐level weight characterization based on the seven subnetworks defined by Yeo et al. [33], along with the subcortical network (Figure 3B). Positive weights predominantly pertained to connections within the visual network, between the frontoparietal and default mode networks, and between the visual and dorsal attention networks. Negative weights primarily involved connections within the somatomotor network, between the somatomotor and default mode networks, as well as between the default mode and ventral attention networks. Furthermore, leveraging the Neurosynth meta‐analytic database [35], we discovered that regions exhibiting greater predictive power within the identified network pattern were predominantly associated with gain‐related processes (Figure 3C).

### 3.5 Testing Model Stability

The weight maps across 82 models exhibited high stability (*r* = 0.94 ± 0.04). Moreover, the average weight map obtained based on the bootstrap test closely resembled that obtained from the PCR model (*r* = 0.85) (Figure S3A). Furthermore, strong concordance was observed between the response network patterns identified by the PCR and PLSR models (*r* = 0.87) (Figure S3B). These findings, examined from multiple perspectives, collectively underscore the stability of the model.

### 3.6 Response Network Strength Compared to HCs

To investigate the relative strength of the response network in HCs compared to patients, we computed the network strength by multiplying the edge weights of the network with the whole‐brain functional connectivity of each individual, encompassing both patients and HCs. Importantly, the HC cohort was entirely independent from the predictive modeling process. No significant difference in network strength was observed between patients and HCs (*t* = −0.52, *p* = 0.60) (Figure S4). However, upon stratifying patients into responders and non‐responders, we discovered that abstinence responders exhibited significantly higher network strength compared to HCs (*t* = 6.03, *p* = 2.5e‐8), whereas non‐responders demonstrated significantly lower network strength than HCs (*t* = −5.56, *p* = 1.9e‐7) (Figure 3D). Additionally, the results remained significant after controlling for gender. Specifically, responders continued to exhibit significantly higher network strength than HCs (*t* = 2.81, *p* = 5.9e‐3), whereas non‐responders continued to show significantly lower network strength relative to HCs (*t* = −3.22, *p* = 1.7e‐3). This suggests that the response networks of responders manifest heightened activity, while the activity of response networks in non‐responders appears to be suppressed.

### 3.7 Binary Prediction

In practical clinical contexts, in addition to predicting continuous treatment outcome metrics, the ability to classify treatment responders and non‐responders at the individual level also holds paramount clinical significance. Therefore, we extended our investigation to assess the efficacy of our identified response network in making binary predictions. In each repetition's test set, we applied a threshold derived from the average network strength observed in the HC cohort for classification purposes: individuals exceeding this threshold were classified as responders, while those below were deemed non‐responders. The results show that the identified response network pattern still showed promise in binary prediction of treatment outcomes (accuracy = 0.60, sensitivity = 0.59 and specificity = 0.61).

### 3.8 Gene Expression Related to the Response Network Pattern

**FIGURE 4:**
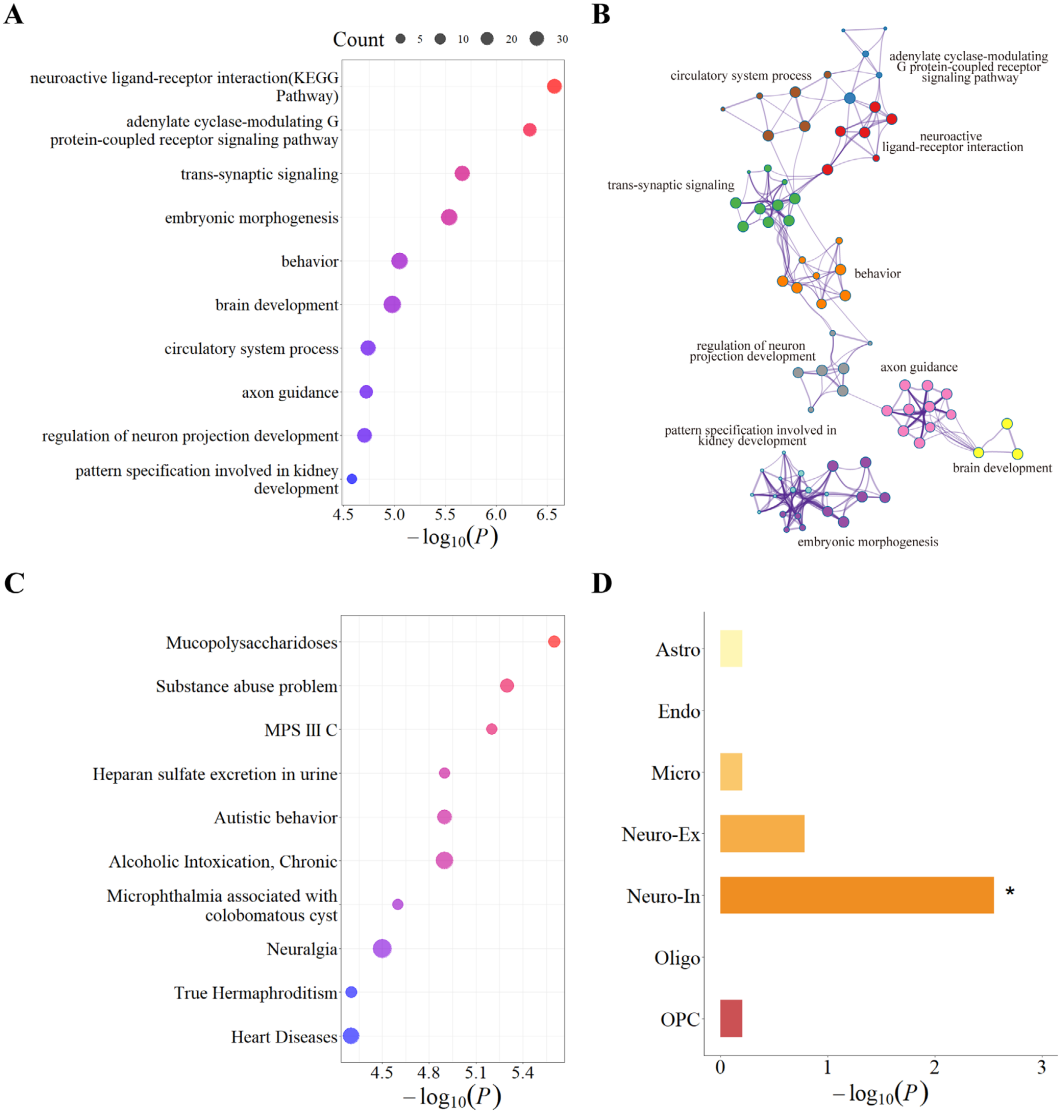
Enrichment analysis for the positive gene list associated with the response network pattern. (A) Ontology terms for the positive gene list. The size of each circle corresponds to the number of genes involved in the given terms. (B) Network visualization of enriched terms with cluster‐based coloring. Each node within the network represents an enriched term, and nodes within the same cluster are colored identically. (C) Enrichment analysis in DisGeNET for the positive gene list. The size of each circle corresponds to the number of genes involved in the given terms. (D) Cell type enrichment analysis. An asterisk denotes *p* value that remained significant after FDR correction (*p* < 0.05). Astro, astrocytes; Endo, endothelial; Micro, microglia; Neuro‐Ex, excitatory neurons; Neuro‐In, inhibitory neurons; Oligo, oligodendrocytes; OPC, oligodendrocyte precursor cells.

To explore the relationship between gene expression and the response network pattern, we conducted association analysis utilizing the AHBA transcriptomic dataset [23]. For each gene, the correlation was determined by assessing the association between its co‐expression matrix and the response network pattern. After sorting genes by correlation coefficient and applying a threshold of 5%, we identified two gene lists: the positive (top 5%, 780 genes) and negative (bottom 5%, 780 genes) gene lists. All gene correlations within both lists were statistically significant (*p*_FDR_ < 0.05). According to the Metascape analysis [39], the top 10 enrichment terms for the positive gene list included KEGG pathways such as “neuroactive ligand‐receptor interaction”, and GO biological processes such as “adenylate cyclase‐modulating G protein‐coupled receptor signaling pathway” and “trans‐synaptic signaling” (Figure 4A,B). Enrichment analysis conducted in DisGeNET indicated that the positive gene list was primarily enriched in substance use disorders (Figure 4C). Furthermore, for the negative gene list, the enriched biological processes encompassed “protein modification by small protein conjugation” and “mRNA metabolic process” (Figure S5A).

We further conducted a cell type enrichment analysis. Genes specific to inhibitory neurons exhibited significant overrepresentation in the positive gene list (*p*_FDR_ < 0.05) (Figure 4D), while genes in the negative gene list were predominantly expressed in oligodendrocytes (*p*_FDR_ < 0.05) (Figure S5B). The results obtained at a threshold of 10% remained essentially stable (Figure S6).

## 4 Discussion

In this study, we demonstrated the ability to use PCR modeling to predict withdrawal responses based on baseline brain functional connectivity patterns. Subsequently, we explored the identified response networks at multiple levels. We then revealed that HCs exhibited moderate differences in network strength between responders and non‐responders. At the same time, we showed that the same network has binarized predictive validity. Finally, we found that the response network pattern was associated with synaptic signaling and inhibitory neurons.

Our results revealed no significant associations between clinical variables and abstinence response scores, suggesting that neuroimaging biomarkers may have earlier predictive value than behavioral measures (e.g., MA use patterns) in differentiating abstinence responders from non‐responders. Future studies with larger sample sizes and extended longitudinal follow‐up durations are warranted to verify these preliminary findings. A FC‐based study on cocaine abstinence outcomes found that baseline clinical characteristics (including years of cocaine use, frequency of use in the past month, and methadone treatment dosage) were not significantly associated with abstinence success. However, FC features significantly predicted cocaine abstinence outcomes [15]. In addition, our study suggests that whole‐brain functional connectivity can effectively predict abstinence response. We discovered that the brain regions associated with positive weights in the prediction model (SFG, MFG, OrG, PhG, and LoCC) and those associated with negative weights (OrG, STG, PCL, pSTS, and CG) were similar to the main brain areas involved in the withdrawal networks of cocaine and opioid addiction [15, 16]. Notably, the OrG contributed to both the positive and negative predictive networks. Because model weights reflect the predictive contribution of distinct functional connections rather than regional activity, this finding may indicate the functional heterogeneity of orbitofrontal circuits. Positive orbital gyrus‐related connections may support adaptive valuation and cognitive‐control processes related to craving reduction, whereas negative connections may reflect persistent reward dysregulation, cue‐related salience, or maladaptive habitual processes associated with poorer abstinence response [41].

MA abstinence was associated with increased within‐network connectivity of the visual network (VIS) and increased between‐network connectivity of frontoparietal network (FPN)–default mode network (DMN) and VIS‐dorsal attention network (DAN), as well as decreased connectivity within somatomotor network (SMN), between SMN and DMN, and between DMN and ventral attention network (VAN). The FPN and DMN networks were found to have high prognostic value for repeated transcranial magnetic stimulation treatment outcomes in individuals with cocaine use disorder [42]. Furthermore, complex networks including SMN, FPN, and DMN were found to play important roles in predicting craving across cocaine and gambling addictions [20]. This finding demonstrates the importance of these networks in predicting treatment response and suggests the possibility that there are common response mechanisms across substance addiction. The FPN and DMN networks play crucial roles in supporting the higher‐order cognitive abilities necessary for the brain's self‐regulation, such as executive control and emotion regulation [43, 44, 45]. These functions are intimately involved in the withdrawal process of individuals with addiction. The SMN is closely associated with the automatization of behavior, which is a key component of the addiction process [46]. After successful withdrawal, drug‐related cues may still trigger strong cravings and automatized responses, which may lead to relapse. Therefore, changes in the SMN may help to reduce the automatization of addictive behaviors and thus improve treatment success. Although the present study adopted a data‐driven predictive approach, the identified response network is consistent with classical addiction theories [47]. Reward‐related and orbital/frontal connections may reflect incentive salience and reward dysregulation, whereas frontoparietal and default mode network connections may support cognitive control and self‐regulation, consistent with the impaired response inhibition and salience attribution framework [48]. The negative contribution of somatomotor‐related connections may further reflect habit‐like or automatized drug‐seeking processes [42]. Thus, the response network may capture distributed neural systems underlying craving persistence and abstinence outcomes.

The brain regions and networks that play a key role in the predictive model partially overlap with the reward circuits, a core mechanism of addiction, and our analysis in conjunction with the Neurosynth meta‐analytic database revealed that these brain regions and networks are associated with reward. Compared to HCs, MA withdrawal responders had higher network strength and showed higher network activity, whereas non‐responders had less network strength. This result suggested a hyperactive response network in abstinent responders versus apparent suppression in non‐responders. The mesolimbic dopamine system, as the canonical reward circuitry, may underlie these observations through its functional alterations [45, 49]. We speculate that the heightened network activity observed in responders relative to HCs likely reflects hyperactivation of the mesolimbic system, which may support successful treatment through compensatory upregulation of dopaminergic and associated neurotransmitter signaling within this pathway [45]. This neuroadaptive response may counteract MA‐induced dopaminergic deficits, consequently reducing drug craving. In contrast, non‐responders may experience persistent reward system suppression with deficient important neurotransmitters such as dopamine. However, because dopamine signaling was not directly measured and the networks were not experimentally manipulated, this interpretation remains associative rather than causal.

Notably, the response networks identified in our study demonstrated both continuous and binary predictive validity. Our findings indicated that the identified networks utilizing the average network strength of the HC cohort as a threshold enabled successful categorical predictions. Identifying treatment responders and non‐responders holds paramount significance in practical clinical settings, particularly within the realm of personalized medicine. This result not only reinforces the observation that HC network strength lies intermediate between responders and non‐responders but also enables prediction at the single‐subject level, which carries important implications for personalized efficacy prediction in clinical practice. From a translational perspective, the identified response network may inform future precision treatment strategies. Brain regions and networks with high predictive contributions, particularly orbital/frontal and frontoparietal‐related circuits, may help prioritize candidate circuits for future individualized neuromodulation studies, rather than directly serving as established stimulation targets. Recent evidence suggests that functional connectivity signatures can be linked to rTMS treatment response in substance use disorders [50]. However, as the present study did not directly test neuromodulation effects, these findings should be considered hypothesis‐generating and require validation in prospective interventional studies. Given the observational and predictive nature of this study, the identified response networks should be interpreted as predictive correlates of abstinence outcomes rather than causal determinants.

In addition, we found that the response network showed enrichment in KEGG pathways (“neuroactive ligand‐receptor interactions”) and GO biological processes (“adenylate cyclase‐modulating G protein‐coupled receptor signaling pathway” and “trans‐synaptic signaling”). Cell‐type enrichment analyses revealed that inhibitory neuron‐specific genes were significantly overexpressed in the positive gene list. These genes showed predominant enrichment in substance use disorders. The KEGG pathway of neuroactive ligand–receptor interaction included neurotransmitter receptor genes related to substance use disorders, such as dopamine, serotonin, γ‐aminobutyric acid, and glutamate receptors, the dysregulation of which was closely associated with addiction [51, 52]. These two GO biological processes participate in regulating chemical synaptic transmission and mediating cellular responses to neurotransmitters, such as “cholinergic synapses”, which play crucial roles in the reward system [53, 54]. A comprehensive genome‐wide study combining alcohol, nicotine, and drug use behavior and disorders also revealed abnormalities in these GO biological processes [55]. Therefore, the better withdrawal effects in MA responders may have been related to the enhancement of these pathways and biological processes. These findings provide preliminary evidence for biological processes related to abstinence‐response prediction and may help inform future investigations of potential therapeutic targets for MA.

Our study has several limitations. Due to the stringent inclusion criteria, which required all participants to voluntarily undergo long‐term abstinence and complete longitudinal follow‐ups, the sample size was relatively small. We excluded participants with comorbid conditions such as anxiety, depression, and sleep disorder by verbal screening, without employing standardized assessment scales. This approach may introduce potential biases in treatment effect analyses due to subjective recall bias or symptom underreporting. In future studies, we plan to implement standardized screening tools to further investigate the efficacy of personalized treatment approaches for individuals with MA use disorders and comorbidities. In addition, our model has not been tested on new MA abstinence subjects. To further validate the reliability of the model, data from MA users at additional sites should be collected and tested. And the efficacy prediction model developed in this study used craving scale score changes during the 1‐year abstinence‐based treatment period as the response indicator. In future research, we will validate the model's predictive validity by collecting actual relapse data through long‐term follow‐up studies. Additionally, future studies should expand the scope to include individuals with other types of substance use disorders to evaluate the applicability of our findings to a broader population of substance use disorders.

This study demonstrated that baseline whole‐brain connectivity holds predictive value for MA abstinence responses. Furthermore, our study revealed that response networks with predictive validity were enriched in biological processes and cell types related to synaptic signaling and inhibitory neurons. These findings enhance our understanding of the multifaceted mechanisms of the abstinence response and provide possible personalized therapeutic strategies and regulatory targets for the treatment of addiction.

## Author Contributions

**Yanyao Du:** writing‐original draft, data curation, software, methodology, formal analysis, funding acquisition. **Shiqi Di:** writing‐original draft, software, methodology, formal analysis. **Na Luo:** methodology, validation, writing‐review and editing. **Wenhan Yang:** data curation, methodology, formal analysis, writing‐review and editing. **Weiyang Shi:** methodology, validation, writing‐review and editing. **Zhengyi Yang:** methodology, validation, writing‐review and editing. **Ming Song:** methodology, validation, writing‐review and editing. **Huiting Zhang:** data curation, software, writing‐review and editing. **Jun Zhang:** data curation, supervision, resources, writing‐review and editing. **Tianzi Jiang:** methodology, project administration, supervision, resources, funding acquisition, writing‐review and editing. **Jun Liu:** methodology, project administration, supervision, resources, funding acquisition, writing‐review and editing.

## Funding

This work was supported by National Natural Science Foundation of China, 82151307, 62327805, 61971451, and U22A20303. STI2030‐Major Projects, 2021ZD0200201. Innovative Province special construction foundation of Hunan Province, 2020SK4001, 2019SK2131. Scientific Project of Zhejiang Lab, 2022ND0AN01, 2022KI0AC02. Graduate Innovation Project of Central South University, 2023XQLH144. Hunan Provincial Graduate Student Research Innovation Program, CX20240306.

## Ethics Statement

This study was approved by the Ethics Committee of the Second Xiangya Hospital of Central South University, Hunan, China (No. 8167071216). Written informed consent was obtained from all participants prior to study participation.

## Conflicts of Interest

The authors declare no conflicts of interest.

## Supporting information


**Table S1:** Brain regions defined by the Brainnetome atlas [1] and corresponding networks [2].
**Table S2:** Demographic Characteristics Between MA and HCs.
**Table S3:** Demographic Characteristics Between MA Responders and HC, and MA Non‐Responders and HC.
**Figure S1:** Residual plot of the predictive model.
**Figure S2:** Predictive performance after eliminating top weighted edges.
**Figure S3:** Predictive pattern stability analysis.
**Figure S4:** Two‐sample t‐test of response network strength between MA and HC.
**Figure S5:** Enrichment analysis for the negative gene list associated with the response network pattern.
**Figure S6:** Enrichment analysis for the positive and negative gene lists derived with a threshold of 10% (comprising 1560 genes in each list).

## Data Availability

The data that support the findings of this study are available from the corresponding author upon reasonable request.
