# Supplementary material for: Functional Brain Network Predictors of Abstinence Treatment Outcomes in Methamphetamine Use Disorder
Source: CNS Neurosci Ther. 2026 Jun 17;32(6):e70990. doi: 10.1002/cns.70990 (PMC13273840; doi:10.1002/cns.70990)
Supplement: Supplementary file 1 — Table S1: Brain regions defined by the Brainnetome atlas [1] and corresponding networks [2]. Table S2: Demographic Characteristics Between MA and HCs. Table S3: Demographic Characteristics Between MA Responders and HC, and MA Non‐Responders and HC. Figure S1: Residual plot of the predictive model. Figure S2: Predictive performance after eliminating top weighted edges. Figure S3: Predictive pattern stability analysis. Figure S4: Two‐sample t‐test of response network strength between MA and HC. Figure S5: Enrichment analysis for the negative gene list associated with the response network pattern. Figure S6: Enrichment analysis for the positive and negative gene lists derived with a threshold of 10% (comprising 1560 genes in each list). [file CNS-32-e70990-s001.docx]

# Supplementary Information

## Supplementary Tables

Table S1. Brain regions defined by the Brainnetome atlas^[1]^ and corresponding networks^[2]^

| Label | Subregion | Region | Network | MNI (X, Y, Z) | Label | Subregion | Region | Network | MNI (X, Y, Z) |
| --- | --- | --- | --- | --- | --- | --- | --- | --- | --- |
| 1 | A8m_l | SFG_L_7_1 | 6 | -5 ,15, 54 | 2 | A8m_r | SFG_R_7_1 | 4 | 7, 16, 54 |
| 3 | A8dl_l | SFG_L_7_2 | 7 | -18, 24, 53 | 4 | A8dl_r | SFG_R_7_2 | 6 | 22, 26, 51 |
| 5 | A9l_l | SFG_L_7_3 | 7 | -11, 49, 40 | 6 | A9l_r | SFG_R_7_3 | 7 | 13, 48, 40 |
| 7 | A6dl_l | SFG_L_7_4 | 3 | -18, -1, 65 | 8 | A6dl_r | SFG_R_7_4 | 3 | 20, 4, 64 |
| 9 | A6m_l | SFG_L_7_5 | 2 | -6, -5, 58 | 10 | A6m_r | SFG_R_7_5 | 2 | 7, -4, 60 |
| 11 | A9m_l | SFG_L_7_6 | 7 | -5, 36, 38 | 12 | A9m_r | SFG_R_7_6 | 6 | 6, 38, 35 |
| 13 | A10m_l | SFG_L_7_7 | 7 | -8, 56, 15 | 14 | A10m_r | SFG_R_7_7 | 7 | 8, 58, 13 |
| 15 | A9/46d_l | MFG_L_7_1 | 4 | -27, 43, 31 | 16 | A9/46d_r | MFG_R_7_1 | 6 | 30, 37, 36 |
| 17 | IFJ_l | MFG_L_7_2 | 6 | -42, 13, 36 | 18 | IFJ_r | MFG_R_7_2 | 6 | 42, 11, 39 |
| 19 | A46_l | MFG_L_7_3 | 6 | -28, 56, 12 | 20 | A46_r | MFG_R_7_3 | 6 | 28, 55, 17 |
| 21 | A9/46v_l | MFG_L_7_4 | 6 | -41, 41, 16 | 22 | A9/46v_r | MFG_R_7_4 | 6 | 42, 44, 14 |
| 23 | A8vl_l | MFG_L_7_5 | 7 | -33, 23, 45 | 24 | A8vl_r | MFG_R_7_5 | 6 | 42, 27, 39 |
| 25 | A6vl_l | MFG_L_7_6 | 3 | -32, 4, 55 | 26 | A6vl_r | MFG_R_7_6 | 3 | 34, 8, 54 |
| 27 | A10l_l | MFG_L_7_7 | 5 | -26, 60, -6 | 28 | A10l_r | MFG_R_7_7 | 6 | 25, 61, -4 |
| 29 | A44d_l | IFG_L_6_1 | 6 | -46, 13, 24 | 30 | A44d_r | IFG_R_6_1 | 3 | 45, 16, 25 |
| 31 | IFS_l | IFG_L_6_2 | 6 | -47, 32, 14 | 32 | IFS_r | IFG_R_6_2 | 6 | 48, 35, 13 |
| 33 | A45c_l | IFG_L_6_3 | 7 | -53, 23, 11 | 34 | A45c_r | IFG_R_6_3 | 7 | 54, 24, 12 |
| 35 | A45r_l | IFG_L_6_4 | 7 | -49, 36, -3 | 36 | A45r_r | IFG_R_6_4 | 6 | 51, 36, -1 |
| 37 | A44op_l | IFG_L_6_5 | 4 | -39, 23, 4 | 38 | A44op_r | IFG_R_6_5 | 4 | 42, 22, 3 |
| 39 | A44v_l | IFG_L_6_6 | 4 | -52, 13, 6 | 40 | A44v_r | IFG_R_6_6 | 4 | 54, 14, 11 |
| 41 | A14m_l | OrG_L_6_1 | 7 | -7, 54, -7 | 42 | A14m_r | OrG_R_6_1 | 7 | 6, 47, -7 |
| 43 | A12/47o_l | OrG_L_6_2 | 7 | -36, 33, -16 | 44 | A12/47o_r | OrG_R_6_2 | 7 | 40, 39, -14 |
| 45 | A11l_l | OrG_L_6_3 | 5 | -23, 38, -18 | 46 | A11l_r | OrG_R_6_3 | 6 | 23, 36, -18 |
| 47 | A11m_l | OrG_L_6_4 | 5 | -6, 52, -19 | 48 | A11m_r | OrG_R_6_4 | 5 | 6, 57, -16 |
| 49 | A13_l | OrG_L_6_5 | 5 | -10, 18, -19 | 50 | A13_r | OrG_R_6_5 | 5 | 9, 20, -19 |
| 51 | A12/47l_l | OrG_L_6_6 | 7 | -41, 32, -9 | 52 | A12/47l_r | OrG_R_6_6 | 7 | 42, 31, -9 |
| 53 | A4hf_l | PrG_L_6_1 | 2 | -49, -8, 39 | 54 | A4hf_r | PrG_R_6_1 | 2 | 55, -2, 33 |
| 55 | A6cdl_l | PrG_L_6_2 | 3 | -32, -9, 58 | 56 | A6cdl_r | PrG_R_6_2 | 3 | 33, -7, 57 |
| 57 | A4ul_l | PrG_L_6_3 | 2 | -26, -25, 63 | 58 | A4ul_r | PrG_R_6_3 | 2 | 34, -19, 59 |
| 59 | A4t_l | PrG_L_6_4 | 2 | -13, -20, 73 | 60 | A4t_r | PrG_R_6_4 | 2 | 15, -22, 71 |
| 61 | A4tl_l | PrG_L_6_5 | 4 | -52, 0, 8 | 62 | A4tl_r | PrG_R_6_5 | 4 | 54, 4, 9 |
| 63 | A6cvl_l | PrG_L_6_6 | 3 | -49, 5, 30 | 64 | A6cvl_r | PrG_R_6_6 | 3 | 51, 7, 30 |
| 65 | A1/2/3ll_l | PCL_L_2_1 | 4 | -8, -38, 58 | 66 | A1/2/3ll_r | PCL_R_2_1 | 2 | 10, -34, 54 |
| 67 | A4ll_l | PCL_L_2_2 | 2 | -4, -23, 61 | 68 | A4ll_r | PCL_R_2_2 | 2 | 5, -21, 61 |
| 69 | A38m_l | STG_L_6_1 | 5 | -32, 14, -34 | 70 | A38m_r | STG_R_6_1 | 5 | 31, 15, -34 |
| 71 | A41/42_l | STG_L_6_2 | 2 | -54, -32, 12 | 72 | A41/42_r | STG_R_6_2 | 2 | 54, -24, 11 |
| 73 | TE1.0/TE1.2_l | STG_L_6_3 | 2 | -50, -11, 1 | 74 | TE1.0/TE1.2_r | STG_R_6_3 | 2 | 51, -4, -1 |
| 75 | A22c_l | STG_L_6_4 | 2 | -62, -33, 7 | 76 | A22c_r | STG_R_6_4 | 2 | 66, -20, 6 |
| 77 | A38l_l | STG_L_6_5 | 5 | -45, 11, -20 | 78 | A38l_r | STG_R_6_5 | 5 | 47, 12, -20 |
| 79 | A22r_l | STG_L_6_6 | 7 | -55, -3, -10 | 80 | A22r_r | STG_R_6_6 | 7 | 56, -12, -5 |
| 81 | A21c_l | MTG_L_4_1 | 7 | -65, -30, -12 | 82 | A21c_r | MTG_R_4_1 | 6 | 65, -29, -13 |
| 83 | A21r_l | MTG_L_4_2 | 7 | -53, 2, -30 | 84 | A21r_r | MTG_R_4_2 | 7 | 51, 6, -32 |
| 85 | A37dl_l | MTG_L_4_3 | 3 | -59, -58, 4 | 86 | A37dl_r | MTG_R_4_3 | 3 | 60, -53, 3 |
| 87 | aSTS_l | MTG_L_4_4 | 7 | -58, -20, -9 | 88 | aSTS_r | MTG_R_4_4 | 7 | 58, -16, -10 |
| 89 | A20iv_l | ITG_L_7_1 | 5 | -45, -26, -27 | 90 | A20iv_r | ITG_R_7_1 | 5 | 46, -14, -33 |
| 91 | A37elv_l | ITG_L_7_2 | 3 | -51, -57, -15 | 92 | A37elv_r | ITG_R_7_2 | 3 | 53, -52, -18 |
| 93 | A20r_l | ITG_L_7_3 | 5 | -43, -2, -41 | 94 | A20r_r | ITG_R_7_3 | 5 | 40, 0, -43 |
| 95 | A20il_l | ITG_L_7_4 | 7 | -56, -16, -28 | 96 | A20il_r | ITG_R_7_4 | 5 | 55, -11, -32 |
| 97 | A37vl_l | ITG_L_7_5 | 3 | -55, -60, -6 | 98 | A37vl_r | ITG_R_7_5 | 3 | 54, -57, -8 |
| 99 | A20cl_l | ITG_L_7_6 | 6 | -59, -42, -16 | 100 | A20cl_r | ITG_R_7_6 | 6 | 61, -40, -17 |
| 101 | A20cv_l | ITG_L_7_7 | 5 | -55, -31, -27 | 102 | A20cv_r | ITG_R_7_7 | 5 | 54, -31, -26 |
| 103 | A20rv_l | FuG_L_3_1 | 5 | -33, -16, -32 | 104 | A20rv_r | FuG_R_3_1 | 5 | 33, -15, -34 |
| 105 | A37mv_l | FuG_L_3_2 | 1 | -31, -64, -14 | 106 | A37mv_r | FuG_R_3_2 | 1 | 31, -62, -14 |
| 107 | A37lv_l | FuG_L_3_3 | 3 | -42, -51, -17 | 108 | A37lv_r | FuG_R_3_3 | 1 | 43, -49, -19 |
| 109 | A35/36r_l | PhG_L_6_1 | 5 | -27, -7, -34 | 110 | A35/36r_r | PhG_R_6_1 | 5 | 28, -8, -33 |
| 111 | A35/36c_l | PhG_L_6_2 | 5 | -25, -25, -26 | 112 | A35/36c_r | PhG_R_6_2 | 1 | 26, -23, -27 |
| 113 | TL_l | PhG_L_6_3 | 1 | -28, -32, -18 | 114 | TL_r | PhG_R_6_3 | 1 | 30, -30, -18 |
| 115 | A28/34_l | PhG_L_6_4 | 5 | -19, -12, -30 | 116 | A28/34_r | PhG_R_6_4 | 5 | 19, -10, -30 |
| 117 | TI_l | PhG_L_6_5 | 5 | -23, 2, -32 | 118 | TI_r | PhG_R_6_5 | 5 | 22, 1, -36 |
| 119 | TH_l | PhG_L_6_6 | 1 | -17, -39, -10 | 120 | TH_r | PhG_R_6_6 | 1 | 19, -36, -11 |
| 121 | rpSTS_l | pSTS_L_2_1 | 7 | -54, -40, 4 | 122 | rpSTS_r | pSTS_R_2_1 | 7 | 53, -37, 3 |
| 123 | cpSTS_l | pSTS_L_2_2 | 4 | -52, -50, 11 | 124 | cpSTS_r | pSTS_R_2_2 | 4 | 57, -40, 12 |
| 125 | A7r_l | SPL_L_5_1 | 3 | -16, -60, 63 | 126 | A7r_r | SPL_R_5_1 | 3 | 19, -57, 65 |
| 127 | A7c_l | SPL_L_5_2 | 3 | -15, -71, 52 | 128 | A7c_r | SPL_R_5_2 | 3 | 19, -69, 54 |
| 129 | A5l_l | SPL_L_5_3 | 3 | -33, -47, 50 | 130 | A5l_r | SPL_R_5_3 | 3 | 35, -42, 54 |
| 131 | A7pc_l | SPL_L_5_4 | 2 | -22, -47, 65 | 132 | A7pc_r | SPL_R_5_4 | 2 | 23, -43, 67 |
| 133 | A7ip_l | SPL_L_5_5 | 3 | -27, -59, 54 | 134 | A7ip_r | SPL_R_5_5 | 3 | 31, -54, 53 |
| 135 | A39c_l | IPL_L_6_1 | 1 | -34, -80, 29 | 136 | A39c_r | IPL_R_6_1 | 1 | 45, -71, 20 |
| 137 | A39rd_l | IPL_L_6_2 | 6 | -38, -61, 46 | 138 | A39rd_r | IPL_R_6_2 | 6 | 39, -65, 44 |
| 139 | A40rd_l | IPL_L_6_3 | 3 | -51, -33, 42 | 140 | A40rd_r | IPL_R_6_3 | 3 | 47, -35, 45 |
| 141 | A40c_l | IPL_L_6_4 | 7 | -56, -49, 38 | 142 | A40c_r | IPL_R_6_4 | 6 | 57, -44, 38 |
| 143 | A39rv_l | IPL_L_6_5 | 3 | -47, -65, 26 | 144 | A39rv_r | IPL_R_6_5 | 7 | 53, -54, 25 |
| 145 | A40rv_l | IPL_L_6_6 | 2 | -53, -31, 23 | 146 | A40rv_r | IPL_R_6_6 | 2 | 55, -26, 26 |
| 147 | A7m_l | PCun_L_4_1 | 6 | -5, -63, 51 | 148 | A7m_r | PCun_R_4_1 | 6 | 6, -65, 51 |
| 149 | A5m_l | PCun_L_4_2 | 2 | -8, -47, 57 | 150 | A5m_r | PCun_R_4_2 | 3 | 7, -47, 58 |
| 151 | dmPOS_l | PCun_L_4_3 | 1 | -12, -67, 25 | 152 | dmPOS_r | PCun_R_4_3 | 1 | 16, -64, 25 |
| 153 | A31_l | PCun_L_4_4 | 7 | -6, -55, 34 | 154 | A31_r | PCun_R_4_4 | 7 | 6, -54, 35 |
| 155 | A1/2/3ulhf_l | PoG_L_4_1 | 2 | -50, -16, 43 | 156 | A1/2/3ulhf_r | PoG_R_4_1 | 2 | 50, -14, 44 |
| 157 | A1/2/3tonIa_l | PoG_L_4_2 | 2 | -56, -14, 16 | 158 | A1/2/3tonIa_r | PoG_R_4_2 | 2 | 56, -10, 15 |
| 159 | A2_l | PoG_L_4_3 | 3 | -46, -30, 50 | 160 | A2_r | PoG_R_4_3 | 2 | 48, -24, 48 |
| 161 | A1/2/3tru_l | PoG_L_4_4 | 2 | -21, -35, 68 | 162 | A1/2/3tru_r | PoG_R_4_4 | 2 | 20, -33, 69 |
| 163 | G_l | INS_L_6_1 | 2 | -36, -20, 10 | 164 | G_r | INS_R_6_1 | 2 | 37, -18, 8 |
| 165 | vIa_l | INS_L_6_2 | 8 | -32, 14, -13 | 166 | vIa_r | INS_R_6_2 | 6 | 33, 14, -13 |
| 167 | dIa_l | INS_L_6_3 | 4 | -34, 18, 1 | 168 | dIa_r | INS_R_6_3 | 4 | 36, 18, 1 |
| 169 | vId/vIg_l | INS_L_6_4 | 4 | -38, -4, -9 | 170 | vId/vIg_r | INS_R_6_4 | 4 | 39, -2, -9 |
| 171 | dIg_l | INS_L_6_5 | 2 | -38, -8, 8 | 172 | dIg_r | INS_R_6_5 | 2 | 39, -7, 8 |
| 173 | dId_l | INS_L_6_6 | 4 | -38, 5, 5 | 174 | dId_r | INS_R_6_6 | 4 | 38, 5, 5 |
| 175 | A23d_l | CG_L_7_1 | 7 | -4, -39, 31 | 176 | A23d_r | CG_R_7_1 | 7 | 4, -37, 32 |
| 177 | A24rv_l | CG_L_7_2 | 8 | -3, 8, 25 | 178 | A24rv_r | CG_R_7_2 | 8 | 5, 22, 12 |
| 179 | A32p_l | CG_L_7_3 | 7 | -6, 34, 21 | 180 | A32p_r | CG_R_7_3 | 4 | 5, 28, 27 |
| 181 | A23v_l | CG_L_7_4 | 7 | -8, -47, 10 | 182 | A23v_r | CG_R_7_4 | 1 | 9, -44, 11 |
| 183 | A24cd_l | CG_L_7_5 | 4 | -5, 7, 37 | 184 | A24cd_r | CG_R_7_5 | 4 | 4, 6, 38 |
| 185 | A23c_l | CG_L_7_6 | 4 | -7, -23, 41 | 186 | A23c_r | CG_R_7_6 | 4 | 6, -20, 40 |
| 187 | A32sg_l | CG_L_7_7 | 7 | -4, 39, -2 | 188 | A32sg_r | CG_R_7_7 | 7 | 5, 41, 6 |
| 189 | cLinG_l | MVOcC_L_5_1 | 1 | -11, -82, -11 | 190 | cLinG_r | MVOcC_R_5_1 | 1 | 10, -85, -9 |
| 191 | rCunG_l | MVOcC_L_5_2 | 1 | -5, -81, 10 | 192 | rCunG_r | MVOcC_R_5_2 | 1 | 7, -76, 11 |
| 193 | cCunG_l | MVOcC_L_5_3 | 1 | -6, -94, 1 | 194 | cCunG_r | MVOcC_R_5_3 | 1 | 8, -90, 12 |
| 195 | rLinG_l | MVOcC_L_5_4 | 1 | -17, -60, -6 | 196 | rLinG_r | MVOcC_R_5_4 | 1 | 18, -60, -7 |
| 197 | vmPOS_l | MVOcC_L_5_5 | 1 | -13, -68, 12 | 198 | vmPOS_r | MVOcC_R_5_5 | 1 | 15, -63, 12 |
| 199 | mOccG_l | LOcC_L_4_1 | 1 | -31, -89, 11 | 200 | mOccG_r | LOcC_R_4_1 | 1 | 34, -86, 11 |
| 201 | V5/MT+_l | LOcC_L_4_2 | 3 | -46, -74, 3 | 202 | V5/MT+_r | LOcC_R_4_2 | 1 | 48, -70, -1 |
| 203 | OPC_l | LOcC_L_4_3 | 1 | -18, -99, 2 | 204 | OPC_r | LOcC_R_4_3 | 1 | 22, -97, 4 |
| 205 | iOccG_l | LOcC_L_4_4 | 1 | -30, -88, -12 | 206 | iOccG_r | LOcC_R_4_4 | 1 | 32, -85, -12 |
| 207 | msOccG_l | LOcC_L_2_1 | 1 | -11, -88, 31 | 208 | msOccG_r | LOcC_R_2_1 | 1 | 16, -85, 34 |
| 209 | lsOccG_l | LOcC_L_2_2 | 1 | -22, -77, 36 | 210 | lsOccG_r | LOcC_R_2_2 | 1 | 29, -75, 36 |
| 211 | mAmyg_l | Amyg_L_2_1 | 8 | -19, -2, -20 | 212 | mAmyg_r | Amyg_R_2_1 | 8 | 19, -2, -19 |
| 213 | lAmyg_l | Amyg_L_2_2 | 8 | -27, -4, -20 | 214 | lAmyg_r | Amyg_R_2_2 | 8 | 28, -3, -20 |
| 215 | rHipp_l | Hipp_L_2_1 | 8 | -22, -14, -19 | 216 | rHipp_r | Hipp_R_2_1 | 8 | 22, -12, -20 |
| 217 | cHipp_l | Hipp_L_2_2 | 8 | -28, -30, -10 | 218 | cHipp_r | Hipp_R_2_2 | 8 | 29, -27, -10 |
| 219 | vCa_l | BG_L_6_1 | 8 | -12, 14, 0 | 220 | vCa_r | BG_R_6_1 | 8 | 15, 14, -2 |
| 221 | GP_l | BG_L_6_2 | 8 | -22, -2, 4 | 222 | GP_r | BG_R_6_2 | 8 | 22, -2, 3 |
| 223 | NAC_l | BG_L_6_3 | 8 | -17, 3, -9 | 224 | NAC_r | BG_R_6_3 | 8 | 15, 8, -9 |
| 225 | vmPu_l | BG_L_6_4 | 8 | -23, 7, -4 | 226 | vmPu_r | BG_R_6_4 | 8 | 22, 8, -1 |
| 227 | dCa_l | BG_L_6_5 | 8 | -14, 2, 16 | 228 | dCa_r | BG_R_6_5 | 8 | 14, 5, 14 |
| 229 | dlPu_l | BG_L_6_6 | 8 | -28, -5, 2 | 230 | dlPu_r | BG_R_6_6 | 8 | 29, -3, 1 |
| 231 | mPFtha_l | Tha_L_8_1 | 8 | -7, -12, 5 | 232 | mPFtha_r | Tha_R_8_1 | 8 | 7, -11, 6 |
| 233 | mPMtha_l | Tha_L_8_2 | 8 | -18, -13, 3 | 234 | mPMtha_r | Tha_R_8_2 | 8 | 12, -14, 1 |
| 235 | Stha_l | Tha_L_8_3 | 8 | -18, -23, 4 | 236 | Stha_r | Tha_R_8_3 | 8 | 18, -22, 3 |
| 237 | rTtha_l | Tha_L_8_4 | 8 | -7, -14, 7 | 238 | rTtha_r | Tha_R_8_4 | 8 | 3, -13, 5 |
| 239 | PPtha_l | Tha_L_8_5 | 8 | -16, -24, 6 | 240 | PPtha_r | Tha_R_8_5 | 8 | 15, -25, 6 |
| 241 | Otha_l | Tha_L_8_6 | 8 | -15, -28, 4 | 242 | Otha_r | Tha_R_8_6 | 8 | 13, -27, 8 |
| 243 | cTtha_l | Tha_L_8_7 | 8 | -12, -22, 13 | 244 | cTtha_r | Tha_R_8_7 | 8 | 10, -14, 14 |
| 245 | lPFtha_l | Tha_L_8_8 | 8 | -11, -14, 2 | 246 | lPFtha_r | Tha_R_8_8 | 8 | 13, -16, 7 |

Network: 1, Visual network; 2, Somatomotor network; 3, Dorsal attention network; 4, Ventral attention network; 5, Limbic network; 6, Frontoparietal network; 7, Default network; 8, Subcortical network.

**Table S2. Demographic Characteristics Between MA and HCs.**

| Characteristic | MA (N=82) | HCs (N=68) | t/χ^2^/Z | *p* |
| --- | --- | --- | --- | --- |
| Age (years) ^a^ | 34.43±8.815 | 40.10±9.51 | -3.787 | <0.001^*^ |
| Gender (male/female) ^b^ | 58/24 | 46/22 | 0.166 | 0.724 |
| Education (years) ^a^ | 9.43±2.61 | 10.90±2.79 | -3.345 | 0.001^*^ |
| Nicotine use (yes/no) ^b^ | 75/7 | 39/29 | 24.696 | <0.001^*^ |
| FTND ^c^ | 5.00(3.00, 6.00) | 3.00(0.00, 5.00) | -4.258 | <0.001^*^ |
| Alcohol use (yes/no) ^b^ | 46/36 | 26/42 | 4.783 | 0.034^*^ |
| AUDI ^c^ | 2.00(0.00, 7.00) | 0.00(0.00, 4.00) | -1.911 | 0.056 |
| Handedness | 82R | 68R | - | - |

^a^ Two-sample t-test. ^b^ Chi-square test. ^c^ Two-sample Wilcoxon-Mann-Whitney U test. MA, methamphetamine user. N, number of subjects. HC, healthy control. FTND, Fagerstrom Test for Nicotine Dependence. AUDIT, Alcohol Use Disorders Identification Test. A significant level was set at *p* < 0.05.

**Table S3. Demographic Characteristics Between MA Responders and HC, and MA Non-Responders and HC.**

| Characteristic | Responders  (N = 39) | Non-responders  (N = 43) | HCs  (N=68) | t/χ^2^/Z^†^ | *p*^†^ | t/χ^2^/Z^#^ | *p*^#^ |
| --- | --- | --- | --- | --- | --- | --- | --- |
| Age (years) ^a^ | 35.75 ± 7.86 | 33.26 ± 9.54 | 40.10±9.51 | -2.439 | 0.016^*^ | -3.688 | <0.001^*^ |
| Gender (male/female) ^b^ | 37/2 | 21/22 | 46/22 | 9.052 | 0.003^*^ | 3.149 | 0.076 |
| Education (years) ^a^ | 9.31 ± 2.33 | 9.53 ± 2.87 | 10.90±2.79 | -3.037 | 0.003^*^ | -2.493 | 0.014^*^ |
| Nicotine use (yes/no) ^b^ | 34/5 | 41/2 | 39/29 | 8.842 | 0.003^*^ | 17.053 | <0.001^*^ |
| FTND ^c^ | 4.49 ± 2.16 | 4.63 ± 2.05 | 3.00(0.00, 5.00) | -3.311 | 0.001^*^ | -3.765 | <0.001^*^ |
| Alcohol use (yes/no) ^b^ | 19/20 | 27/16 | 26/42 | 1.113 | 0.291 | 5.420 | 0.020^*^ |
| AUDI ^c^ | 0.00 (0.00, 6.00) | 3.00 (0.00, 8.00) | 0.00(0.00, 4.00) | -0.897 | 0.370 | 2.250 | 0.024^*^ |
| Handedness | 39 R | 43 R | 68R | - | - | - | - |

^a^ Two-sample t-test. ^b^ Chi-square test. ^c^ Two-sample Wilcoxon-Mann-Whitney U test. ^†^Statistical values for the comparison between MA responders and HCs. ^#^Statistical values for the comparison between MA non-responders and HCs. MA, methamphetamine user. N, number of subjects. HCs, healthy controls. FTND, Fagerstrom Test for Nicotine Dependence. AUDIT, Alcohol Use Disorders Identification Test. A significant level was set at *p* < 0.05.

## Supplementary Figures

**Figure S1. Residual plot of the predictive model.**

**
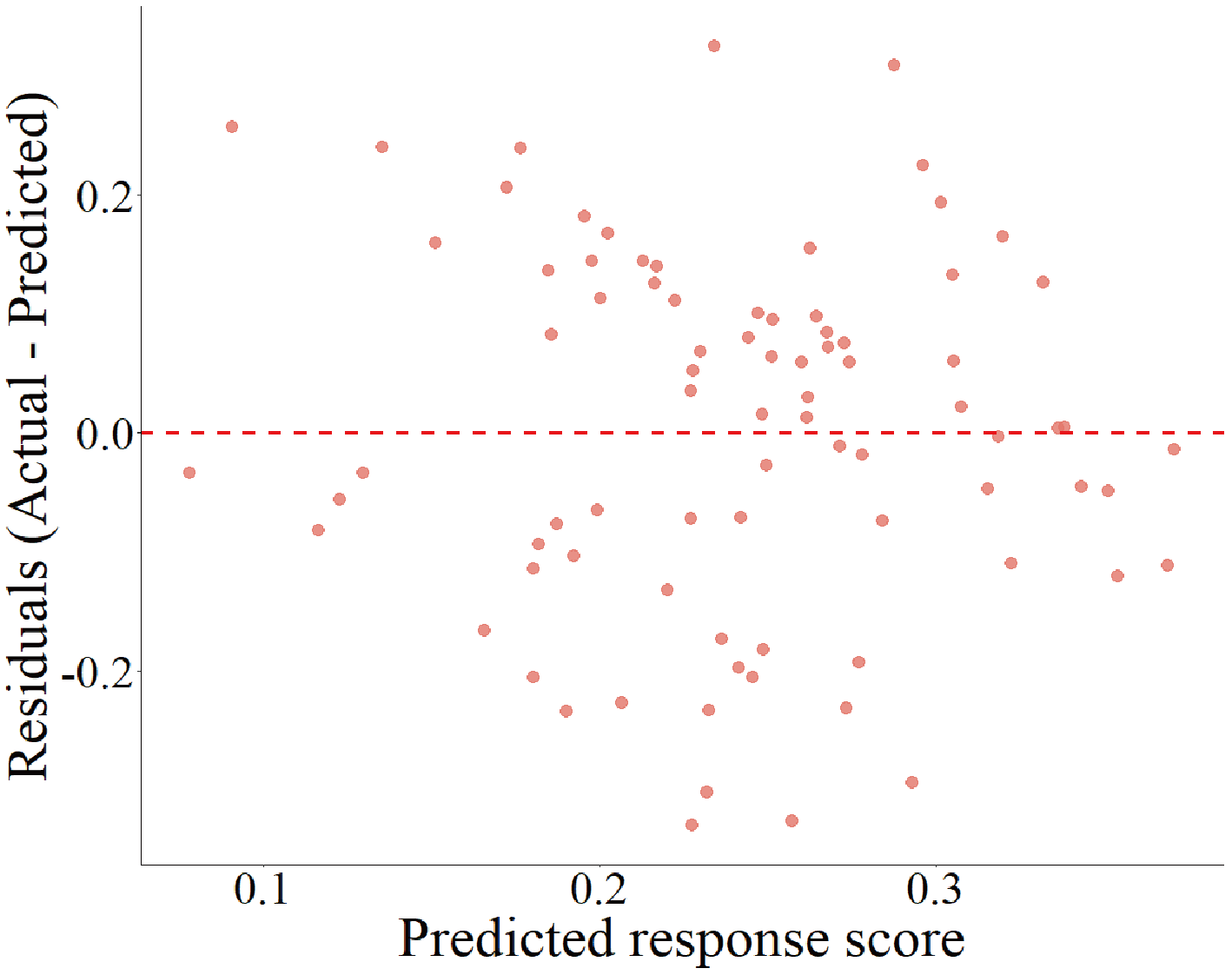
**

Residual plot of the predictive model. Residuals were randomly distributed around zero, indicating no obvious systematic bias. The mean residual was close to zero (mean residual = 0.0014), no significant heteroscedasticity was detected using the Breusch–Pagan test (*p* = 0.22), and no extreme outliers were identified, further supporting the robustness of the predictive model.

**Figure S2. Predictive performance after eliminating top weighted edges.**
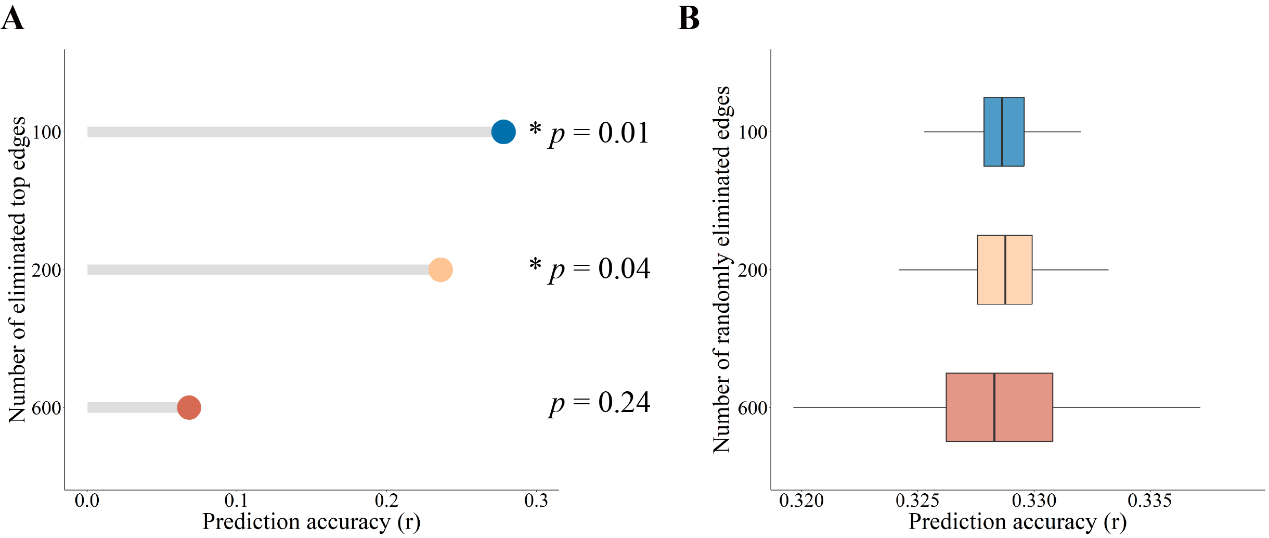


(A) Prediction accuracies after eliminating top weighted edges in both the positive and negative networks. The top 50, 100, and 300 edges in the positive and negative networks, corresponding to edge numbers of 100, 200, and 600, respectively, were eliminated. After removing the top 300 edges in both networks, the remaining edges were unable to predict the response scores (*r* = 0.07, *p* = 0.24). (B) The distribution of prediction accuracies derived from randomly eliminating 100, 200, and 600 edges 1000 times each. Randomly removing the same number of edges as in (A) did not lead to a degradation in prediction performance.

**Figure S3. Predictive pattern stability analysis.**
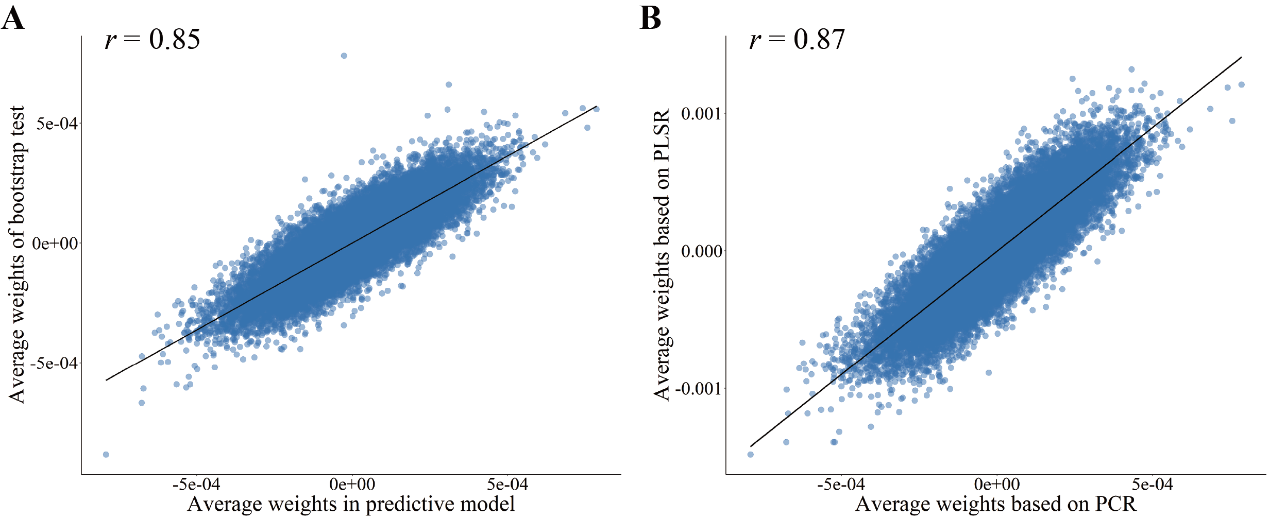


(A) The correlation between the average weights derived from the PCR models and those derived from 5000 bootstrap test iterations. (B) The correlation between the average weights derived from the PCR models and those obtained from the PLSR models. PCR, principal component regression. PLSR, partial least squares regression.

**Figure S4. Two-sample t-test of response network strength between MA and HC.**


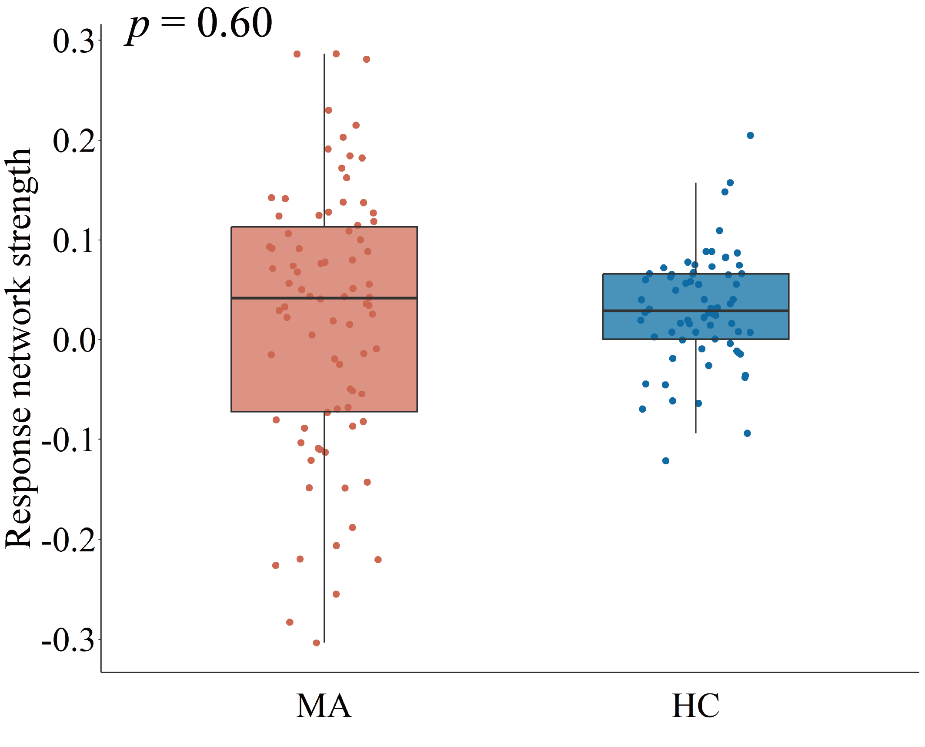


MA, methamphetamine group. HC, healthy control group.

**Figure S5. Enrichment analysis for the negative gene list associated with the response network pattern.**
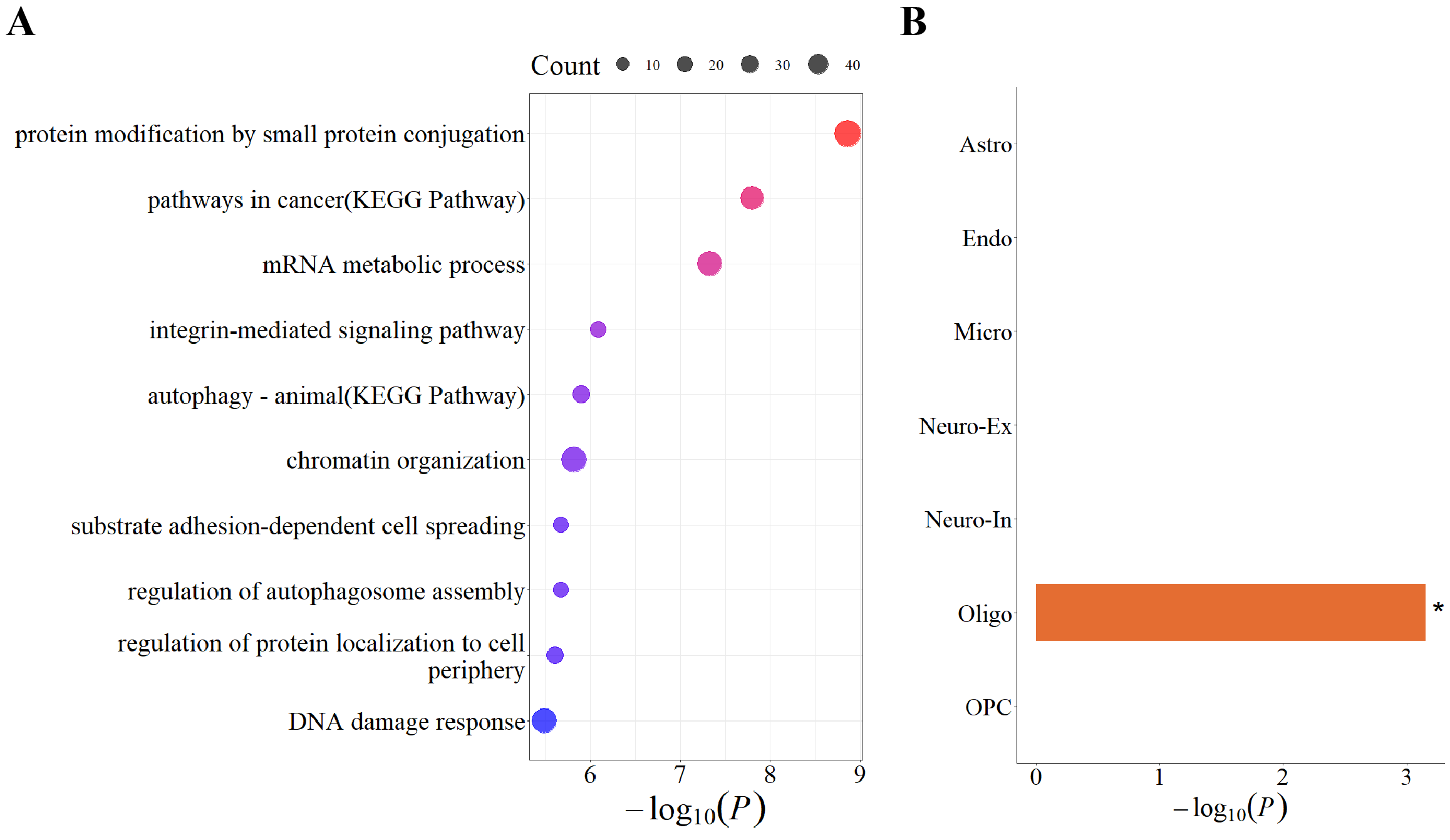


(A) Ontology terms for the negative gene list. The size of each circle corresponds to the number of genes involved in the given terms. (B) Cell type enrichment analysis. An asterisk denotes p value that remained significant after FDR correction (*p* < 0.05). Astro, astrocytes; Endo, endothelial; Micro, microglia; Neuro-Ex, excitatory neurons; Neuro-In, inhibitory neurons; Oligo, oligodendrocytes; OPC, oligodendrocyte precursor cells.

**Figure S6. Enrichment analysis for the positive and negative gene lists derived with a threshold of 10% (comprising 1560 genes in each list).**


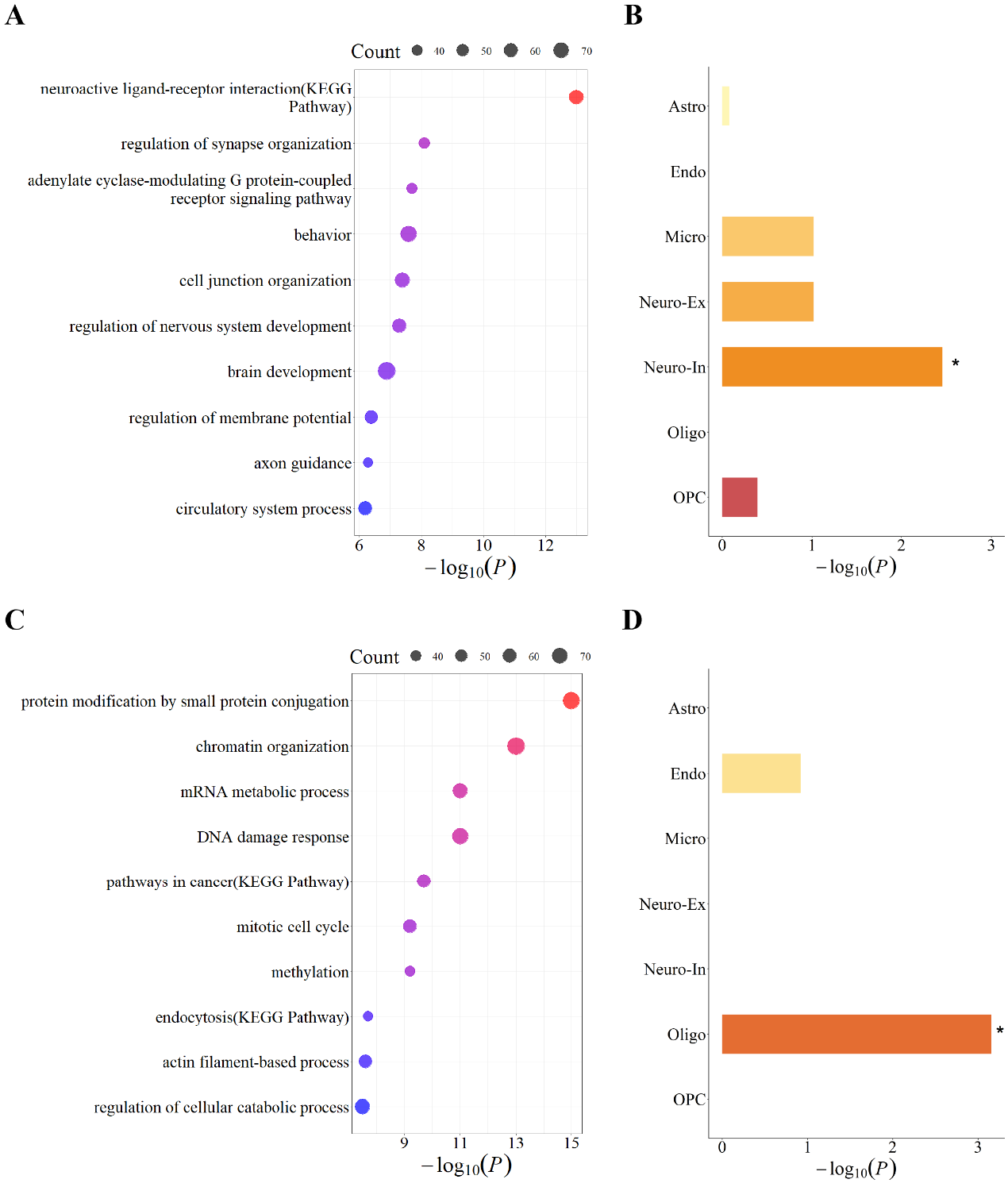


(A) Ontology terms for the positive gene list. (B) Cell type enrichment analysis for the positive gene list. (C) Ontology terms for the negative gene list. The size of each circle corresponds to the number of genes involved in the given terms. (D) Cell type enrichment analysis for the negative gene list. An asterisk denotes p value that remained significant after FDR correction (*p* < 0.05). Astro, astrocytes; Endo, endothelial; Micro, microglia; Neuro-Ex, excitatory neurons; Neuro-In, inhibitory neurons; Oligo, oligodendrocytes; OPC, oligodendrocyte precursor cells.

## Supplementary references

[1] Fan L, Li H, Zhuo J, et al. The Human Brainnetome Atlas: A New Brain Atlas Based on Connectional Architecture[J]. Cerebral Cortex (New York, N.Y.: 1991), 2016, 26(8): 3508-3526.

[2] Yeo B T T, Krienen F M, Sepulcre J, et al. The organization of the human cerebral cortex estimated by intrinsic functional connectivity[J]. Journal of Neurophysiology, 2011, 106(3): 1125-1165.
